# Parental awareness and knowledge of first aid for children in Saudi Arabia: a multiregional cross-sectional study

**DOI:** 10.3389/fped.2025.1575783

**Published:** 2025-05-23

**Authors:** Ola Alotaibi, Sarah Alrayya, Shahad Alotaibi, Yara Algoraini

**Affiliations:** ^1^ Pediatric Department, King Fahad Medical City, Riyadh, Saudi Arabia; ^2^ Emergency Department, King Khalid University Hospital, Riyadh, Saudi Arabia; ^3^ Pediatric Emergency Department, King Fahad Medical City, Riyadh, Saudi Arabia

**Keywords:** pediatric, first aid, awareness, knowledge, Saudi Arabia

## Abstract

**Background:**

First aid (FA) knowledge is crucial for parents to effectively respond to pediatric emergencies, and it can potentially save lives and reduce injury severity. Despite the high rates of childhood accidents such as burns, choking, and drowning, limited data exist on parental awareness of FA in Saudi Arabia across various situations. The aim of this study was to assess parental knowledge and awareness regarding FA for children in Saudi Arabia.

**Methods:**

This cross-sectional study using a self-administered electronic questionnaire was conducted via social networking sites between March and April 2024. The questionnaire was developed from a literature review and validated by experts. A total of 1,421 parents aged ≥18 years and residing in Saudi Arabia participated. Data were analyzed using descriptive statistics and logistic regression analysis.

**Results:**

The participants were predominantly women (59.3%), Saudi nationals (85.1%), married (90.6%), and university graduates (68.3%). While 73.6% believed they had good FA knowledge, only 25.6% had attended a formal FA course. With regard to knowledge gaps, 44.12% parents incorrectly believed that they should encourage a choking child aged <1 year to cough, while 61.44% incorrectly believed that ice should be applied to burns; only 51.02% parents knew that cool water should be run over burns. Logistic regression analysis showed that non-Saudi nationality and previous FA experience were significant predictors of willingness to attend FA courses (*p* = 0.045 and *p* = 0.012, respectively). Barriers to FA knowledge included underestimation of the problem (39.3%) and lack of information from healthcare personnel (28.1%). Preferred methods for gaining FA knowledge were inclusion of FA in educational curricula (59.7%) and programs in primary healthcare centers (19.5%).

**Conclusion:**

There are significant gaps in parental knowledge regarding FA for children in Saudi Arabia. These gaps need to be addressed through structured educational programs and accessible resources in order to enhance preparedness for pediatric emergencies among parents. Recommendations include implementation of FA training programs, integration of FA into school curricula, development of educational materials in Arabic, and promotion of mandatory FA training during prenatal and postnatal care visits.

## Background

In today's fast-paced world, knowledge of first aid (FA) techniques can make the difference between life and death, especially when it comes to the safety and well-being of children. FA was defined by the American Heart Association (AHA) and the American Red Cross (ARC) in 2010 as “assessments and interventions that can be performed by a bystander or by the victim with minimal or no medical equipment” (1, 2). FA is performed to provide immediate assistance to an injured or sick individual until medical professionals arrive. Beyond basic life support, it encompasses interventions designed to prolong life, alleviate distress, prevent further harm or illness, and expedite injury recovery.

Early childhood is an essential phase in the growth of a child, and young children are very susceptible to accidents because their bodies are still developing and they cannot recognize and various threats from the environment by themselves. Motor development in the beginning stages places children at risk of injury due to exploration and interaction with the world, the lack of complete physical coordination of their musculature, and immature depth perception (3). Moreover, accidental injuries due to incidents such as burning, choking, and drowning have the potential to occur inside residential settings and educational institutions catering to young children. A Spanish study investigated the degree of familiarity with FA among primary and preschool teachers as well as the parents of the children. The findings revealed that 57% participants understood FA. However, only four individuals could define the basic life support sequence, while none managed to respond accurately to questions related to cardiopulmonary resuscitation (CPR) (4). Another study conducted in Saudi Arabia (SA) assessed the knowledge and attitudes of mothers with regard to FA for their children; the results revealed that approximately 65.5% and 69.8% participating mothers held inaccurate beliefs regarding the definition of FA and its components, respectively. In addition, approximately 67.4% were misinformed about FA for burns (5). A recent investigation on the pattern of accidental injuries in an emergency department (ED) involving children aged <14 years in Abha City, SA revealed that pediatric trauma was a significant concern in the Aseer region (6). Moreover, in 94% cases, mothers played the role of primary caregivers. The study concluded that it is essential to educate mothers regarding preventative measures to effectively reduce the incidence of accidental injuries, particularly those involving foreign bodies (6). Most injuries in preschool require only FA. Given the frequency of accidents requiring FA, preschools should emphasize on injury and infection prevention measures for children. A study conducted in Royal Children's Hospital, Australia documented the clinical outcomes of all patients with acute burn injuries who presented to a children's hospital and received FA which revealed, the majority of patients utilized cold water as first aid (80.2%), although only 12.1% adhered to the suggested duration of 20 min or more. Nonetheless, recommended FA (cold water for ≥20 min) was linked to a markedly decreased reepithelialization duration for children with contact injuries (*P* = .011) (7, 8). Because of the frequency of pregnant mothers and family members administering FA at home and in preschools, it is essential to understand the beliefs of parents and the general public about accidents in children (9).

Numerous researchers worldwide have investigated the extent of FA awareness among various population groups (4, 10). However, studies on parental knowledge and awareness regarding FA for children are limited in SA and the Gulf region (5, 6). The aim of this study was to assess the level of parental knowledge and awareness regarding FA for children in Saudi Arabia.

## Methods

### Study design

This study employed a cross-sectional design using a self-administered electronic questionnaire to assess parental awareness and knowledge of pediatric first aid in Saudi Arabia.

### Questionnaire development and validation

The questionnaire was developed following an extensive literature review and adaptation of items from previously published studies (10, 11). Face validity was ensured by three independent experts, and pilot testing was conducted to refine the questionnaire, reduce ambiguity, and confirm clarity. To accommodate the diverse language preferences of participants, the instrument was prepared in both Arabic and English using a backward–forward translation method.

### Pilot study

Prior to the main survey, a pilot study was conducted with a convenience sample of 50 parents (aged ≥18 years and residents of Saudi Arabia) to evaluate the questionnaire's clarity, cultural appropriateness, and overall comprehensiveness. The objectives were to identify ambiguities in wording, assess the clarity of instructions, and determine the time required to complete the survey. Participants completed the questionnaire and provided structured feedback on question wording, potential misunderstandings, and overall survey length. Based on the pilot results, several items were reworded to eliminate ambiguity, redundant questions were removed, and adjustments were made to ensure that the language was both accessible and culturally relevant. These refinements enhanced the reliability and validity of the final questionnaire used in the main study.

### Data collection procedure

The survey was distributed via social media platforms (e.g., WhatsApp and Twitter) between March and April 2024. Participants were required to be aged ≥18 years and residents of Saudi Arabia. Informed consent was obtained implicitly when participants opted to complete the questionnaire after reading an introductory note outlining the study's objectives and procedures.

### Sample size determination

Assuming a 50% awareness rate and a 3% margin of error at a 95% confidence interval, the minimum sample size calculated using Cochran's formula was 1,068. To enhance reliability, the sample size was increased to 1,421 participants.

### Data management and statistical analysis

Data were analyzed using SPSS version 25.0. Descriptive statistics summarized the demographic characteristics and levels of first aid awareness. To identify predictors of willingness to attend first aid courses, logistic regression analysis (using a stepwise descending method) was conducted. This analytical approach was selected because it is well-suited for modeling binary outcomes and evaluating the association between multiple predictors and the likelihood of engaging in first aid training, which aligns directly with our study's objectives. Supporting literature emphasizes the utility of logistic regression in similar cross-sectional designs (10, 11).

To ensure the adequacy of our regression model, we evaluated several model fitness criteria. The Hosmer–Lemeshow goodness-of-fit test yielded a *p*-value of 0.45, indicating a good fit between the model and the observed data. The Nagelkerke *R*^2^ value was 0.25, suggesting that approximately 25% of the variance in willingness to attend a first aid course was explained by the model. Furthermore, the overall classification accuracy was 78%, further supporting the adequacy of our logistic regression model.

## Results

### Participant demographics

In total, 1,421 parents who responded to the questionnaire were included in this study. Their average age was 38 ± 10 years (Table 1), and the majority were women (59.3%), while 85.1% were Saudi nationals. With regard to the marital status, 90.6% participants were married. Maximum participants were from the central region (41.8%), followed by the southern region (16.6%). Most participants (68.3%) had a university education, with only 3.0% having less than high school education.

**Table 1:** Parents sociodemographic characteristics.

Characteristics	Description	*N* = 1,421 (%)
Age (year)	Min–max	0–81
	Mean ± SD	38 ± 10
	Median (P25–P75)	36 (31–45)
Gender	Female	843 (59.3)
	Male	578 (40.7)
Nationality	Non-Saudi	212 (14.9)
	Saudi	1,209 (85.1)
Marital Status	Divorced	45 (3.2)
	Widower	88 (6.2)
	Married	1,288 (90.6)
State of Residence	Southern region	236 (16.6)
	Eastern region	169 (11.9)
	Northern region	219 (15.4)
	Western region	203 (14.3)
	Central region	594 (41.8)
Educational Level	Less than high school	43 (3.0)
	Illiterate	3 (0.2)
	High School	205 (14.4)
	University	970 (68.3)
	Post grad. (Master, PhD)	200 (14.1)
Your Professional background is?	Student	23 (1.6)
	Unemployed	480 (33.8)
	Retired	95 (6.7)
	Employed	823 (57.9)
How many children do you have?	min–max	0–12
	Mean ± SD	3 ± 2
	Median	2 (1–3)
Children age range	1 Years–3 years	322 (22.6)
	6 years–12 years	311 (21.8)
	12 Years–18 years	247 (17.3)
	3 Years–6 years	245 (17.2)
	1 month–1 year	168 (11.8)
	1 day–1 month	128 (9)
What's your family monthly income?	<6,000 SAR	508 (35.7)
	6,000–12,000 SAR	449 (31.6)
	>12,000 SAR	464 (32.7)

### Awareness and perceived knowledge of first aid

Most parents (85.79%) had heard the term FA, while 34.34% had read a book or leaflet about it (Table 2). Despite this, only 63.12% knew the Saudi Red Crescent phone number, highlighting a gap in key emergency information. When questioned about their degree of knowledge, 73.55% parents believed they had good knowledge of pediatric FA, with 12.53% rating their knowledge as excellent. However, 13.93% reported having poor knowledge. Practical experience in FA was limited, with only 32.09% parents having provided FA before and 59.61% having encountered situations that required it (Table 3).

**Table 2:** Parents’ awareness about first Aid and their sources of information.

Characteristics	Description	*N* = 1,421 (%)
How well is your knowledge about pediatric first aid?	Good	1,045 (73.55)
	Poor	198 (13.93)
	Excellent	178 (12.53)
Did you ever face cases that needed first aid?	Yes	847 (59.61)
	No	574 (40.39)
Have you Ever provided first aid care?	Yes	456 (32.09)
	No	965 (67.91)
Have you Ever heard about the word first aid?	Yes	1,219 (85.79)
	No	202 (14.22)
Do you Know the Saudi Red Crescent phone number?	Yes	897 (63.12)
	No	524 (36.88)
Have you Ever Read a book or leaflet about first aid?	Yes	488 (34.34)
	No	933 (65.66)
Source of information* (*n* = 1,219)	Mass Media/Social media	936 (65.87)
	Family/Friends	187 (13.16)
	School/University	96 (6.76)

* More than one answer was allowed.

**Table 3:** Parents history of training in first-aid course.

Characteristics	Description	*N* = 1,421 (%)
Did you attended a first aid course before?	Yes	363 (25.55)
	No	1,058 (74.45)
If (Yes) The Feeling of confidence in managing incidents needing first aid for your children	Somewhat	150 (10.55)
	Not confident	113 (7.95)
	Very confident	100 (7.04)
Do you Know where to attend for first aid course?	Yes	495 (34.84)
	No	926 (65.17)
Are you willing to attend a first aid course	Yes	1,178 (82.9)
	No	243 (17.1)
If your answer to the previous question is (No) please specify why?* (*n* = 243)	No enough time	118 (49.17)
	Difficulty in Transportations	44 (18.11)
	Financial issues	29 (11.93)
	Work-related issues	23 (9.46)
	Not interested	15 (6.17)
	Others (as There are no suitable place, elderly, attended before, afraid to apply it in real situations)	14 (5.76)

* More than one answer was allowed.

### First aid training and confidence

Among those who had attended an FA course (25.55%), confidence levels for managing incidents requiring FA were mixed: 7.04% felt very confident, 10.55% were somewhat confident, and 7.95% were not confident. Despite these gaps in knowledge and experience, 82.9% parents expressed a willingness to attend an FA course.

### Specific knowledge of first aid practices

Evaluation of specific knowledge regarding FA in various emergency situations revealed important gaps. For choking in children <1 year old, 65.31% parents knew that they should not insert their fingers into a choking child's mouth, and 62.98% knew that they should not hang the child upside down, whereas 44.12% believed they should not encourage their <1 year old child to cough (Table 4). For burns, 61.44% parents incorrectly believed that ice should be applied to burns, whereas only 51.02% knew that they should run cool water over the burn. For drowning, 68.88% correctly identified the recovery position for an unresponsive but breathing child. With regard to seizures, 72.84% parents were aware that they should not insert objects into a child's mouth, while 47.99% knew that they should place the child on their side to aid breathing. For epistaxis, nearly half the participants (49.89%) knew that the child should bend forward and avoid tilting the head backward.

**Table 4:** Parents knowledge about first-aid for Various incidents.

Statement	Correct knowledge *n* (%)
A-CHOKING for <1 years old child*	
Slap him on the back	799 (56.23)
Not to encourage him/her to cough and to take a deep breath	627 (44.12)
Not to hang them upside down by their feet	895 (62.98)
Not to insert fingers into the victim mouth looking for the toy and trying to remove it	928 (65.31)
B-BURN*	
Not to place ice on the burn	548 (38.56)
Not to place honey over the affected area	978 (68.82)
Put the affected area under cool running water until the pain is relived	725 (51.02)
Remove clothing adherent to the affected skin	753 (52.99)
Use a proper burn ointment	875 (61.58)
Not to wrap a clean towel moistened with cold water until the pain of the burns subsides	635 (44.69)
C-DROWNING for unresponsive but breathing child*	
Put him on a left lateral position (recovery position)	979 (68.88)
Not to start a chest compression	965 (67.91)
Remove his clothes	599 (42.15)
Cover him with your jacket or sweater	803 (56.52)
Not to lye him on his back (supine position)	743 (52.29)
Not to shake him vigorously to try wake him	961 (67.64)
D-SIZURES*	
Not to put any object in the mouth of the injured person	1,035 (72.84)
Stay with the injured person until the seizure ends	942 (66.3)
Gently place the victim on one side to help him breathe	682 (47.99)
Secure the area around him and make sure that there is nothing around him that could harm him	622 (43.77)
Not to try to stop the movements during the seizure	615 (43.28)
E-EPISTAXIS*	
Apply pressure on the nose for at least 5 min	671 (47.22)
Bend slightly forward while sitting or standing, and avoid lying down or tilting the head backwards	709 (49.89)
Warm compresses can not be placed on the nose	459 (32.3)

* More than one answer were allowed.

### Predictors of willingness to attend first aid courses

Logistic regression analysis identified key predictors for parents’ willingness to attend FA courses (Table 5). Nationality was a significant predictor, with non-Saudis more likely to attend courses (*p* = 0.045). In addition, parents with previous experience in providing FA were significantly more willing to attend future courses (*p* = 0.012).

**Table 5:** Logistic regression analysis of positive predicting factors of whom willing to attend first-Aid course. (n-1,178).

Feature	95% CI		Odds ratio	*P*-value
	Lower	Upper		
Age	−0.001892	0.00767	1.000917	0.23
Gender (Female)	−0.005621	0.007455	1.150243	0.78
Nationality (Saudi)	0.003198	0.297288	0.915569	0.04*
Marital Status (Married)	−0.185432	0.01657	1.004906	0.09
Educational Level (University)	−0.082345	0.050685	1.017215	0.65
Professional Background (Employed)	−0.027894	0.062324	0.999432	0.45
Number of Children	−0.007123	0.005987	0.988469	0.87
Children Age Ranges (1 Years–3 years)	−0.035123	0.012061	0.949463	0.34
Monthly Income (<6,000 SAR)	−0.113456	0.012382	0.954265	0.12
Faced First Aid Cases (Yes)	−0.121432	0.029962	1.113552	0.23
Provided First Aid Care (No)	0.025678	0.201426	0.975018	0.01*
Read First Aid Book (No)	−0.032167	0.092659	1.000917	0.34

* *P*-value <0.05 statistically significant.

In the logistic regression analysis, the outcome variable “willingness to attend a first aid course” (categorized as “Yes” vs. “No”) was examined against several predictors (Table 5). Two variables emerged as statistically significant. Saudi Nationality was significant (*p* = 0.04) with an odds ratio of 0.92 (95% CI: 0.0032, 0.2973), indicating that differences in nationality were associated with varying levels of willingness to attend a course. Additionally, the variable “provided first aid care” was significant (*p* = 0.01) with an odds ratio of 0.98 (95% CI: 0.0257, 0.2014), suggesting that parents who had previously provided first aid care were more likely to be willing to attend a course. Other predictors, including age, gender, marital status, educational level, professional background, number of children, children's age range, monthly income, having faced first aid cases, and reading a first aid book, did not show statistically significant associations with the outcome. These findings highlight the potential impact of nationality and previous first aid experience on parents’ willingness to engage in further training.

### Barriers and preferred educational methods

**Figure 1:**
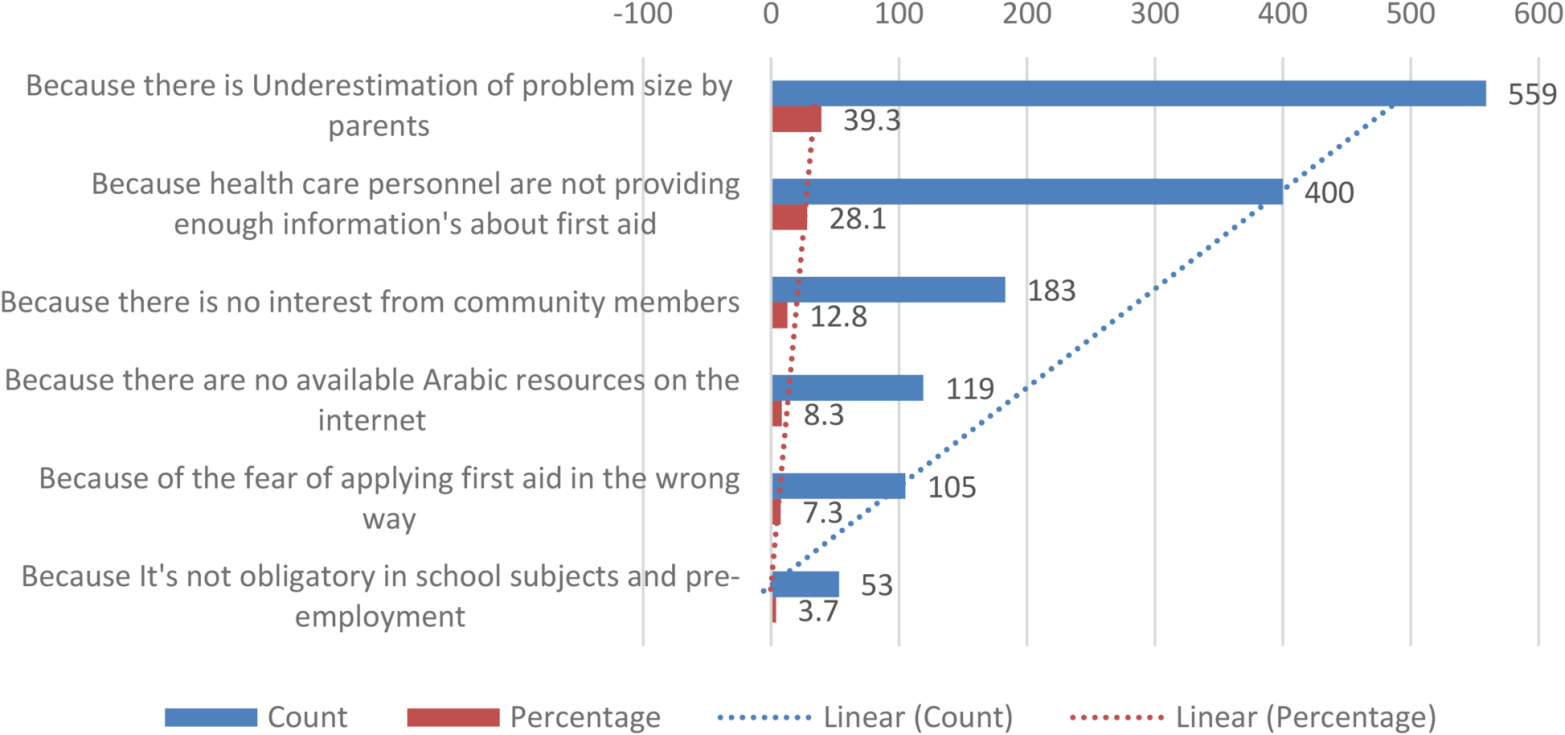
Reasons of parents lacking of knowledge in first-aid.

**Figure 2:**
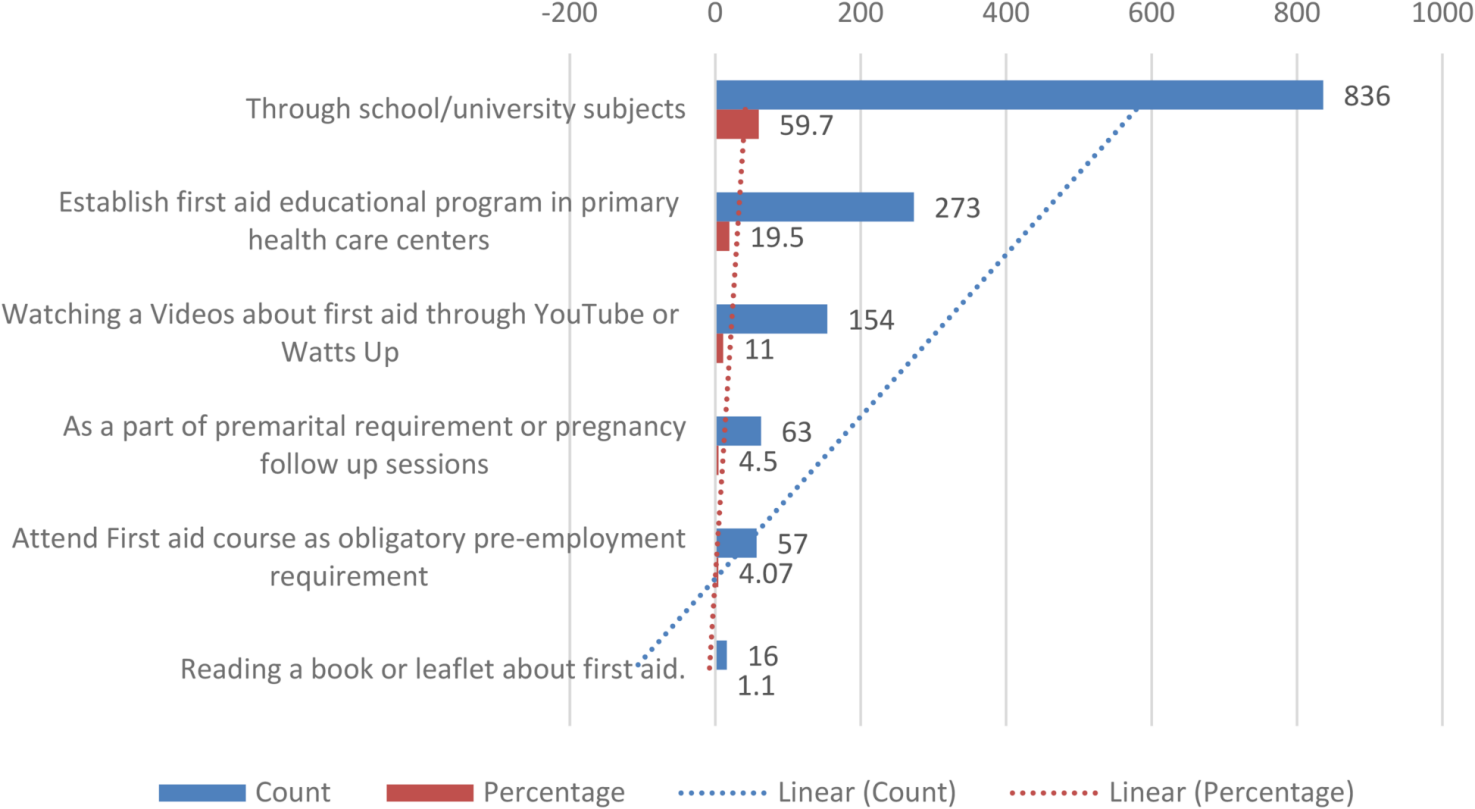
Parents recommendations about best Way to gain knowledge in first-aid.

Figure 1 highlights barriers to acquiring first aid knowledge; 39.3% parents cited underestimation of the problem as a key factor, while 28.1% attributed their lack of knowledge to the lack of information from healthcare personnel. When asked about preferred methods for gaining first aid knowledge, 59.7% parents suggested incorporation of first aid education into school/university curricula, while 19.5% advocated for the establishment of educational programs in primary healthcare centers (Figure 2).

These results underscore the importance of enhancing FA training programs for parents in SA, as significant gaps in both knowledge and practical experience were observed.

## Discussion

FA knowledge among parents is crucial for them to handle emergencies that children face from time to time more effectively and promptly, thus enabling them to protect their children's health and even saving their life (12, 13). In SA, there is a concerning increase in the incidence of injuries among children, including those caused by burns, falls, and choking (14, 15). Considering the cultural beliefs and practices deeply rooted in this population (16), we question whether misconceptions surrounding these practices influence ways in which parents address emergencies faced by their children. In the present study, we found significant gaps in specific FA knowledge, highlighting the need for improved educational initiatives. The majority of participating parents were women (59.3%) and Saudi nationals (85.1%) with a mean age of 38 ± 10 years. Most were married (90.6%) and had graduated from university (68.3%). These demographics are consistent with those in previous studies conducted in Saudi Arabia; thus, our sample was representative of the general parent population in the country (17, 18).

Although 73.55% parents rated their knowledge of pediatric FA as “good,” only 25.55% had ever attended an FA course. Similar findings were reported in a study conducted in Riyadh, where only 18% parents had received formal FA training (19). A study conducted in Jeddah, SA by Gajendran emphasized the importance of developing FA training and skills, which are crucial for handling emergency health situations. The study reiterated that students need to be equipped with sufficient practice and knowledge to manage illnesses and injuries common in a school setting (20).

Studies from the United States and Canada have also highlighted inadequate FA knowledge among parents, emphasizing the global nature of this issue (21, 22).

Mass media and social media were the primary sources of FA-related information in this study (65.87%). While these platforms can disseminate information rapidly, the accuracy of the content is not always reliable. A study in Canada found that misinformation on social media could lead to improper FA practices among parents (23). This reliance on informal sources may contribute to the misconceptions observed in the present study. Similar findings were reported in a study from SA (24, 25).

Our findings largely align with previous research conducted in Saudi Arabia, which reports notable gaps in parental FA knowledge. For example, Al-Johani et al. (24) observed that a significant proportion of parents lacked formal FA training, a finding similar to our own observation of only 25.55% of participants having attended an FA course. However, subtle differences in reported percentages suggest that varying cultural contexts, sample demographics, and methodologies may influence these outcomes. For instance, while a study from Riyadh reported an even lower training rate of 18% (19), a study in Jeddah by Gajendran (20) highlighted the importance of FA skills, indicating that regional differences might affect parental attitudes and the accessibility of training programs.

Studies from North America also contribute a contrasting perspective. Research in the United States by Murphy et al. (21) and in Canada by Kendrick et al. (22) revealed that although many parents felt confident about their FA abilities, their actual knowledge and recent training updates were considerably lacking. These findings imply that self-assessment of FA proficiency may not accurately reflect practical competence, a point that is corroborated by our results where a high percentage of parents rated their FA knowledge as good, despite low formal training attendance.

When assessing specific FA knowledge for various incidents, several gaps were identified. Choking is the primary cause of unintentional injury-related fatalities in children <1 year of age (26), and appropriate FA knowledge is essential to respond rapidly in such circumstances. An alarming 34.69% parents believed they should insert their fingers into the child's mouth to remove the object, not knowing that this action can push the obstruction further down the airway. Similar misconceptions have been reported in the United States and SA, where parents lacked proper knowledge regarding the management of choking infants (11, 24, 27).

Burn injuries are considered among the most catastrophic injuries and represent the fourth most prevalent type of trauma globally (28). In the present study, only 51.02% parents knew that they should run cool water over the affected area, which is the recommended immediate response (28). Misconceptions such as application of honey (31.18%) or ice (61.44%) were prevalent, with similar findings in other studies from SA (11, 24). Similarly, a study conducted by Batais et al. in SA observed that although a considerable proportion of the participants reported having personal experience with burns and had received some information regarding FA for burns, only about half could correctly provide responses about FA techniques (29). Furthermore, a study conducted by Al-Bshri and Jahan in Buraidah, Qassim, SA reported inadequate knowledge and practice of FA among parents, with a substantial proportion (58.2%) of participants demonstrating only fair knowledge (30). In Australia, a study found that only 9% parents provided adequate FA treatment for burns, indicating a widespread lack of proper knowledge (8).

Drowning ranks as the third most common cause of accidental injury-related fatality (31, 32). While 68.88% parents in the present study knew how to place the child in the recovery position, 32.09% incorrectly believed that initiation of chest compressions in an unresponsive but breathing child was necessary, with similar findings in another study from SA (11). This confusion about CPR protocols has also been observed in other countries (33, 34).

With regard to seizures, a minority of parents (27.16%) incorrectly believed that they should place an object in the child's mouth; this is a dangerous myth that persists globally (24, 35). Similar misconceptions were observed in a study by Habbash et al., who reported that beliefs in spirit possession and the evil eye are embedded in Saudi culture; only 25% and 19% parents reported receiving information about seizures from doctors and seminars, respectively (36). A Saudi study conducted by Al-Dosary et al. to assess public awareness of FA measures for seizures found that the overall awareness rate was inadequate at only 57.5% (37). This highlighted the importance of social media and field training campaigns to increase awareness regarding FA for seizures**.** Educational campaigns in the United Kingdom have aimed to dispel such myths, although challenges persist (38).

Epistaxis is a concerning condition that occurs commonly in children, either spontaneously or as a result of trauma. Nevertheless, the majority of parents lack enough understanding regarding FA for epistaxis. Less than half the parents in the present study knew the correct management techniques, consistent with the findings in a study conducted by Alam et al., who reported that 30.9% participants demonstrated a good level of knowledge regarding FA for epistaxis (39). Additionally, a study conducted by Mahzara et al. reported that about 60% participants had experienced epistaxis; however, only 52% had received prior FA training (40), revealing notable gaps in knowledge and practices of FA for epistaxis among the general population in the Jazan region, SA. In the present study, only 47.22% applied pressure on the nose while 49.89% recognized the importance of leaning forward, with similar findings in another study from SA (24). Incorrect practices like tilting the head back are common worldwide and can exacerbate the condition (24, 41–42).

Our findings are consistent with those of previous research conducted both within SA and internationally, highlighting gaps in parental knowledge regarding pediatric FA. In SA, studies have reported similar deficiencies in parental FA knowledge. A study conducted in Riyadh found that parents lacked adequate knowledge to manage common pediatric emergencies, particularly burns and choking incidents (43, 44).

In the United States, research has shown that while many parents feel confident in handling emergencies, their actual FA knowledge is inadequate. The ARC (45) reported that only 15% parents had recently updated their FA skills, indicating the need for continuous education. Similarly, in Canada, studies have indicated that parents have limited practical knowledge for managing pediatric emergencies despite general awareness of FA concepts.

Logistic regression analysis in the present study identified that non-Saudi nationals were more willing to attend an FA course (*p* = 0.045).Moreover, parents who had previously provided FA were more inclined to seek training (*p* = 0.012). This suggests that personal experience in handling emergencies motivates parents to acquire formal skills, a trend observed in studies from the United States and Canada (46).

We found that primary reasons for the lack of FA knowledge included underestimation of the problem by parents (39.3%) and insufficient information provided by healthcare personnel (28.1%). A study in Jeddah reported similar barriers, emphasizing the need for healthcare providers to play a more active role in parental education (47). Fear of performing FA incorrectly (7.3%) reflects a lack of confidence that can be mitigated through practical training (48, 49).

Moreover, while several studies support the benefits of integrating FA into educational curricula—as evidenced by the “Kids Save Lives” campaign in the United States (33) and mandatory school-based programs in parts of Europe (50)—other research underscores potential pitfalls. Gupta et al. (23) discussed how misinformation on social media might lead to improper FA practices, a concern that contrasts with our findings where mass media was a predominant information source. This discrepancy suggests that the quality of information disseminated through social platforms may vary significantly across different regions and populations.

In summary, although the overall trends in parental FA knowledge are similar across various studies, the differences in percentages and associations indicate that multiple factors—such as cultural norms, socioeconomic status, regional training accessibility, and the reliability of information sources—play critical roles in shaping FA practices. These nuanced differences call for further investigation to better understand the diverse pathways influencing FA knowledge and to tailor educational interventions accordingly.

### Limitations

First, this study was cross-sectional which involved an online questionnaire instead of face-to-face interviews; this may have resulted in bias as selection bias can occur as individuals without internet access or digital literacy may be excluded, leading to underrepresentation of certain demographics. Also, response bias may result from participants providing rushed or less accurate answers due to the absence of personal interaction. Additionally, the lack of an interviewer to clarify questions can cause misunderstandings or incomplete responses. These factors highlight the limitations of online surveys compared to in-person interviews. The reliance on digital platforms may have introduced selection bias, excluding individuals with limited internet access or lower digital literacy. This limitation could have led to an overrepresentation of participants who are more technologically adept and possibly more engaged or informed about first aid. Additionally, the self-reported nature of the data may have introduced response bias, where participants might overestimate their knowledge or willingness to attend first aid courses due to social desirability. Such biases could affect the generalizability of our findings. In future studies, incorporating mixed-method approaches, such as face-to-face interviews or practical assessments, may help to validate and extend these results. Second, although we used a representative sample size, there were variations in the number of collected responses. Moreover, more responses were obtained from certain regions than from others. The highest response rate was noted for the central region, as Riyadh is one of the largest cities in the country. This could mean that individuals in this region are more aware of and concerned about FA-related issues. Other factors may have played a role, such as Internet accessibility and regional variances in attitudes regarding the use of social media. Finally, there may be some ambiguities in translation, whereby words or phrases in English did not translate correctly into Arabic. Future research should include objective assessments of FA skills and explore the effectiveness of educational interventions over time.

## Conclusions

There is a clear need to improve FA knowledge among parents in SA. By addressing the identified gaps through education and training, it is possible to enhance the preparedness of parents to effectively manage pediatric emergencies, ultimately improving child health outcomes. To address the identified gaps in parental first aid knowledge, we recommend a multifaceted approach that includes integrating FA training into school curricula. This integration could be achieved by forming partnerships between educational authorities and local healthcare organizations to develop standardized training modules tailored to different age groups. Pilot programs in select schools can be used to refine these modules, ensuring that the training is culturally appropriate and practically applicable. Regular, hands-on practice sessions, coupled with periodic evaluations, would ensure sustained competency and readiness to handle pediatric emergencies. Such an approach not only makes FA education more accessible but also supports long-term public health objectives by fostering a community well-prepared to respond effectively in emergency situations. FA training for parents should be mandatory during prenatal and postnatal care visits, as implemented in some regions in the US and Canada (9, 51). Further longitudinal studies should assess the effectiveness of educational interventions for improving FA knowledge and outcomes.

## Data Availability

The original contributions presented in the study are included in the article/Supplementary Material, further inquiries can be directed to the corresponding author.
